# Effect of Anchote (*Coccinia abyssinica*) and Potato Starch Addition on Colloidal Stability of Pineapple Juice

**DOI:** 10.1155/2021/6615273

**Published:** 2021-05-03

**Authors:** Abebaw Teshome Tiruneh, Abebaw Ayele Negatu, Neela Satheesh

**Affiliations:** Faculty of Chemical and Food Engineering, Bahir Dar Institute of Technology, Bahir Dar University, P. O. Box 26, Ethiopia

## Abstract

Starch is one of the most important value-added food ingredients used as a thickener in many foods and industrial applications. This research investigated the effect of different concentrations of starch (anchote and potato) addition on the colloidal stability of pineapple juice. The experiment was carried out on a two-factor factorial design arranged in CRD. The first factor (starch type with two levels (anchote and potato)) and the second factor (starch concentration with three levels (1%, 3%, and 5%)) were considered. The starch-added juice samples were preserved for 15 days at room temperature. The physicochemical properties, colloidal stability, microbial counts, and sensory analysis were conducted in a 7-day interval including the first day. The results revealed that different starch concentrations showed a significant effect (*P* ≤ 0.05) on the cloud stability, most of the physicochemical properties and microbial count of pineapple juice as compared to the control. The turbidity and viscosity of the juice samples were increased significantly (*P* ≤ 0.05) by the starch addition; in contrast, pulp sedimentation and microbial counts were decreased. As storage duration increased, turbidity, viscosity, TSS, pH, and vitamin C content of juice decreased, whereas sedimentation, TA, and microbial count increased. The results revealed that the total bacterial and fungal counts of pineapple juice samples were rising as storage durations increased. The maximum cloud retention was observed in juice added with 5% anchote starch. Finally, it is confirmed that starch (anchote and potato) addition positively affected the colloidal stability of pineapple juice and also possessed high potential to extend the shelf life.

## 1. Introduction

Pineapple (*Ananas comosus (L.) Merr.*) is the most versatile, commercially important, nonclimacteric tropical fruit that belongs to the Bromeliaceae family [1–3]. Pineapple fruit is a rich source of sugars, fiber, organic acids, minerals (calcium, iron, and potassium), and vitamins (A, B, and C). Half a cup of pineapple juice provides 50% of an adult's daily recommended amount of vitamin C [4]. Pineapple contains bromelain, a proteolytic enzyme, which is highly useful in protein degradation [2]. Due to the good flavor, aroma, juiciness, and sweetness, pineapple fruits are well known and appreciated by consumers throughout the world. In addition, pineapple is also a rich source of health-promoting antioxidants, such as ascorbic acid, flavonoids, and other phenolic compounds; hence, it is attracting both the consumer and processor [2, 5–7]. Pineapple fruits are widely consumed in fresh or as the processed juice among all the age groups. Pineapple juice is classified as a nonalcoholic beverage, and the demand is rising constantly due to the consumer awareness on its health benefits [8, 9].

Pineapple is a seasonal fruit crop and perishable in nature, and due to the presence of high sugar and moisture contents, postharvest losses during peak harvesting seasons are considerably high (40%) [2, 3]. Hence, alternative processing and preservation methods are very important [10]. Pineapples can preserve for a short duration in cold storage further, processed into shelf-stable value-added products (juice, desserts, squash, jam, jelly, and canned pineapple slices) to reduce postharvest losses [6, 11]. Pineapple juice is an unstable suspension that settles quickly after extraction, and such phase separation depreciates the visual appearance of the product [11].

A small percentage of insoluble particles (mixture of proteins, pectins, lipids, hemicellulose, cellulose, and other minor components) remain in the cloudy juice processing. Achieving the bright, natural color is not possible in cloudy fruit juices [12–14]. In addition, cloud loss in juices is an objectionable scenario to the consumers. Presence of insoluble matter in clear beverages is considered as an indication of spoilage. On the other hand, colloidal suspensions in fruit juices are preferred, as cloud imparts characteristic flavor, color, and mouth feel. Therefore, it is important to maintain and improve juice turbidity to meet consumer demands [14, 15].

Hydrocolloids are used widely in fruits, vegetables, and protein-based juices to improve color or cloud stability for prolonged periods due to their thickening (raising the viscosity) and suspension properties [11, 14–17]. Starch is increasingly used as a functional ingredient in industrial applications and food processing due to its ability as a thickening agent [18].

Hence, using starch as a stabilizer in fruit juice proves to be vigorous due to its effectiveness, availability, and low in cost. On the other hand, starch does not alter the organoleptic and sensory properties of the juice compared to other hydrocolloids. Researchers reported the use of carboxymethyl cellulose (CMC), low-methoxyl pectin, guar, xanthan, and gellan gum, [16, 19], sodium alginate, [14], and guar gum [15, 17, 20] in carrot, orange, mulberry, and apple juices. Starch is widely used in yogurt preparation as a thickener to reduce defects, making the body and texture of manufactured yogurt appealing as well as reducing cracks in the surface of the curd milk [18, 21]. However, studies on starch addition in fruit juice for colloidal stability are relatively limited. So, in this study, we used different concentrations of the starch from potato and anchote as the stabilizer in pineapple juice.

Anchote (*Coccinia abyssinica*) is an indigenous root tuber crop widely produced in southern and southwestern parts of Ethiopia. Anchote is a drought tolerant crop providing food security; it is a highly productive and nutritionally ample crop [22]. Nutritionally, anchote is a good source of carbohydrates, proteins, minerals, and fiber [22, 23]. Potato is also an important ingredient for nutrition due to the good source of starch, vitamins A and C, and minerals such as iron and potassium and fiber [18].

The objective of this research is to determine the effect of potato and anchote starch at 1%, 3%, and 5% on the physiochemical properties, microbial growth, and stability of pineapple juice during short storage durations at room temperatures.

## 2. Materials and Methods

### 2.1. Procurement of the Ingredients

Fully ripened pineapple (Cayenne cultivar) was purchased from the local market of the Bahir Dar City. Anchote (*Coccinia abyssinica*) tuber was collected from the local market of Nekemte Town, Oromia Region, Ethiopia. Commercial potato starch was purchased from the local market of Bahir Dar City, Ethiopia.

### 2.2. Starch Extraction from Anchote

As the anchote starch was not readily available, isolation and purification were done by Sit et al. [24] method. The raw anchote tuber was washed and was peeled and chopped using a mechanical blender for 4 min (EP 5, vertical cutter-mixer, France). A 10% (*w*/*v*) crushed tuber mass and water suspension were prepared. The suspension was filtered through 250 *μ*m sieves, and the filtrate was allowed to settle for 12 h. The supernatant was decanted, and the sediment was washed until the pure white in color is obtained. The resulting starch was finally dried at room temperature, milled to fine powder in a mortar and pestle, sieved through 224 *μ*m mesh, and stored in an airtight plastic container under dry conditions for further use [22].

### 2.3. Experimental Design

The experiment was set as 2 × 3 factorial design arranged in CRD. The first factor considered starch type with two levels (potato and anchote). The second factor is starch concentration in three levels (1, 3, and 5%), and each treatment was conducted in triplicate and total experimental runs conducted in this experiment were 18 and pineapple juice without any starch addition was considered as control.

### 2.4. Preparation of Pineapple Juice

The fully ripened pineapple fruits were washed thoroughly with potable water to remove dirt. The cleaned pineapples were peeled off and cut into pieces with sterile stainless steel kitchen knife. Then, the pineapple pieces were homogenized in a clean electric laboratory disperser (SWFS1.1-00, China). The pineapple juice was filtered through 500 *μ*m aperture stainless steel sieve screen into a clean transparent plastic bowl [7, 25]. Then, commercial potato and anchote starches were added to the pineapple juice at concentration levels of 1%, 3%, and 5% (*w*/*v*) as a designed experiment. The juice was mixed by a magnetic stirrer until complete dissolution of the starch. After incorporation of the starch, pineapple juices were bottled and allowed for storage studies at room temperature (28°C ± 2°C) for fifteen (15) days. The stability of color and cloud was measured at every 7-day interval for 15 days.

### 2.5. Determination of Physicochemical and Functional Properties of Starch

#### 2.5.1. Bulk Density

Bulk density of starch samples was determined according to the method of Ohizua et al. [26]. Starch sample (50 g) was taken into a 100 ml measuring cylinder. The cylinder was tapped several times on a laboratory bench to a constant volume. Bulk density (g/cm^3^) was calculated by dividing the weight of sample on its volume after tapping.

#### 2.5.2. Water Absorption Capacity (WAC**)**

The WAC of the starch samples was determined by the method of Bello-Pérez et al. [27]. One gram of flour sample was mixed with 10 ml of distilled water and allowed to stand at ambient temperature (30 ± 2°*C*) for 30 min, and then centrifuged (L-530 tabletop low-speed centrifuge, China) for 30 min at 3000 rpm. The clear supernatant was decanted. Water absorption was expressed as percent water bound per gram flour.

#### 2.5.3. Swelling Power (SP) and Water Solubility Index (WSI)

Swelling power and WSI were determined according to the method described by Bello-Pérez et al. [27]. 0.5 g of the starch sample was taken in a preweighed centrifuge tube. About 10 ml of distilled water was added and mixed gently. The tubes were heated in a thermostatic water bath at 20, 50, 65, 75, and 85°C for 30 min by shaking every 5 min interval and then cooled to room temperature. The suspensions were centrifuged for 15 min at 2000 rpm. The supernatants were decanted immediately after centrifugation to preweighed Petri dishes and dried in an oven for 2 h at 120°C. The residues obtained after drying of the supernatant represent the amount of starch solubilized in water. The solubility was calculated as gram per 100 g of sample on a dry weight basis. The sediment obtained was weighed to determine the swelling of the starch. The swelling power and water solubility index were determined according to the following equations:

(1) $$\begin{array}{c} \text{Swelling power}=\frac{\text{Weight of swollen granules}\times 100}{\text{Weight of sample}-\text{Weight of dissolved starch}}, \end{array}$$

(2) $$\begin{array}{c} \text{Solubility index}\left(\%\right)=\frac{\text{Weight of dried starch in Petri dish}\times 100}{\text{Sample weight}}. \end{array}$$

### 2.6. Determination of Proximate Composition of Potato and Anchote Starch

The moisture content, ash, crude protein, and fat contents were determined according to the AOAC (2006) method numbers 950.46, 920.153, 992.15, and 989.05, respectively. The total starch content was determined by the AOAC (2007) method number 996.11 [22].

### 2.7. Determination of Amylose and Amylopectin Content of Potato and Anchote Starch

Amylose and amylopectin contents were determined by using the method of Hassan and Hassan [28] as cited by Williams et al. [29]. Briefly, 0.10 g of the sample was weighed into a 100 ml volumetric flask and 1 ml of 99% ethanol and 9 ml of 1 M sodium hydroxide solution was added. The contents were mixed thoroughly and heated for 10 min in boiling water (100°C) to gelatinize the starch. After cooling, the solution was made up to the calibration mark with distilled water and shaken thoroughly. 5 ml of prepared starch solution was taken into a 100 ml volumetric flask and was treated with 1 ml of 1 M acetic acid and 2 ml of iodine solution. The solution was diluted to the calibration mark with distilled water, and the absorbance was determined by a spectrophotometer at 620 nm. Amylose and amylopectin contents were calculated using the following equations.

(3) $$\begin{array}{c} \text{Amylose content}\left(\%\right)=3.06\times \text{absorbance}\times 20, \end{array}$$

(4) $$\begin{array}{c} \text{Amylopectin}\left(\%\right)=100-\%\text{amylose content}. \end{array}$$

### 2.8. Determination of Physicochemical Properties of Pineapple Juice

#### 2.8.1. Total Soluble Solids

Total soluble solid (TSS) content of pineapple juice was measured using a hand refractometer (RX-5000i-Plus, Atago, Tokyo, Japan) (AOAC 2000). 1 ml of a well-homogenized pineapple juice was placed on the prism of a calibrated hand refractometer. The readings were taken, and results were expressed in °Brix.

#### 2.8.2. pH and Titratable Acidity

pH of starch and pineapple juice was determined by using a calibrated pH meter with standard solution of pH 4 and 7. Each sample (10 ml) was taken into a beaker, and then, electrodes of the pH meter (PHS-25/3C, China) were immersed into the sample and the readings were recorded directly (AOAC, 2004).

The titratable acidity (TA) was determined according to AOAC (2004) procedure. 10 ml of sample was taken into a conical flask, and three drops of phenolphthalein indicator were added. The mixture was titrated against 0.1 N NaOH solution. The TA was calculated by the standard formula by using a citric acid titer value and expressed as the citric acid [30].

#### 2.8.3. Viscosity

Dynamic viscosity of starch and pineapple juice was determined in an Ostwald viscometer (VISCO STAR+H, Spain) at 20°C and expressed in mPa s [14, 31].

#### 2.8.4. Turbidity

The stability of pineapple juice was assessed by serum cloudiness (turbidity). Cloud stability of the centrifuged samples (4000 rpm for 15 min) (expressed in terms of % light transmission) was determined by measuring absorbance at 660 nm using a UV-Vis spectrophotometer (Agilent Cary 60 UV-Vis spectrophotometer, USA) calibrated with distilled water. The absorbance at 660 nm was directly related to the turbidity of pineapple juice and expressed in nephelometric turbidity units [11, 15, 16].

#### 2.8.5. Sedimentation Measurement

The pulp sedimentation of pineapple juice was determined according to the method of Silva et al. [11]. The pineapple juice sample was transferred into a graduated 100 ml cylinder and stored at 25°C for 24 h (early evaluation) and for a total of 15 days (simulating a shelf life evaluation). The volume of sediment was measured (total volume minus the serum phase), and the sedimentation index was calculated as follows:

(5) $$\begin{array}{c} \text{IS}=\frac{V_{\inf}}{V_{\text{total}}}, \end{array}$$

where *V*_inf_ is the sediment volume (ml) and *V*_total_ is the total volume of the sample (ml).

### 2.9. Vitamin C Content

Vitamin C content was determined by an iodometric titration method as described by Nweze et al. [32].

### 2.10. Sensory Acceptability

Sensory acceptability of pineapple juice was conducted by untrained 20 volunteer panelists (8 females and 12 males) in a hedonic test. The coded pineapple juice samples were presented to panelists randomly for likeness scores on sensory evaluation (taste, flavor, color, consistency, aroma, texture, mouth feel, and overall acceptability) by using a seven-point hedonic rating scale where 7 represents like extremely and 1 represents dislike extremely [1, 33].

### 2.11. Shelf Life Determination of the Pineapple Juice

The pineapple juices with different starch levels were stored at ambient temperature for fifteen (15) days. The shelf life of the juice was determined by checking the pH weekly and by determining the microbial growth [9].

#### 2.11.1. Microbiological Analysis of Pineapple Juices

Enumerations of aerobic plate count (APC) and total fungal counts were performed according to the ISO standard method (ISO-4833:2003(E)). Enumeration of APC was done by the serial dilution technique followed by a pour plate method.

At the initial day, 7^th^ day, and 15^th^ day, serial dilution was carried out by taking pineapple juice from each bottle. The serially diluted juice sample (0.1 ml) with dilution factor 10^3^ for fungal enumeration and 10^4^ for bacterial enumeration was plated and incubated at 37°C for 24 h for bacteria and for fungi at room temperature for 72 h. The colonies from bacteria were counted using the colony counter. The mean value of the triplicate was taken, and the number of colonies was multiplied by the dilution factor and calculated as 1 ml of original sample. It was then expressed as colony-forming unit per ml (cfu/ml) of the sample [9].

### 2.12. Statistical Data Analysis

A triplicate data was subjected to analysis of variance (ANOVA) using Minitab version 19.2. Analysis of variance was performed with the general linear model. The mean separation was done by the Tukey method and considered a significant difference at *P* ≤ 0.05. The results were presented as the mean ± standard deviation.

## 3. Results and Discussions

### 3.1. Physicochemical and Functional Properties of Starch

The results on physicochemical and functional properties of the potato and anchote starches are given in Table 1. Previous studies report that blends of starches have been used as thickening agents and stabilizers to control water mobility, facilitate processing, and improve stability in food systems [18, 34].

A significant difference (*P* ≤ 0.05) was observed in the functional properties of potato and anchote starch except in bulk density and pH. The bulk densities of potato and anchote starches were observed as 1.5 g/ml and 1.4 g/ml, respectively. The bulk density is affected by the particle size and the density of the starch flour. This functional property is very important in determining the packaging requirements, material handling, and the compatibility in wet processing in food industry [35]. The higher bulk density of starch suggests their suitability for use in food preparations. In contrast, low bulk density would be an advantage in the formulation of complementary foods [36]. The present study revealed that bulk density of starches was observed higher; it indicates that starch would serve as better thickeners in food products.

The mean WAC of 1.4 g/g and 1.1 g/g was observed for potato and anchote starch, respectively. The highest WAC of potato starch could be attributed to the presence of higher amount of carbohydrates (starch) and fiber. The fibers have good ability to associate with water under limited water presence (high hydration properties) [35]. The WAC is the ability of the starch to hold water against gravity. Presence of proteins and carbohydrates enhances the WAC of starch by providing hydrophilic groups like polar and charged side chains. Water plays an important role in food quality and stability. The WAC is desirable in different foods to imbibe water without dissolution of protein, thereby attaining body thickening and viscosity [37]. High water holding capacity will rise to high swelling power and high peak viscosity. Root and tuber starches with high level of WAC are useful in meeting the needs for starch incorporation into juice production [38].

The SP showed 4.9% for potato starch, whereas 6.2% for anchote starch. The capacity to hydrate and swelling allows changes in starch viscosity. The higher amylopectin content is responsible for higher SP and viscosity at low temperatures. The swelling capacity of starch granules allows increasing their viscosity and gelling properties [39]. The differences in SP of the two starches may be ascribed to variations in amylose and nonstarch contents. The swelling power showed weak negative correlation with amylose content. This suggests that higher swelling starches had lower amylose and protein contents [40].

The WSI values of the starches were 1.2 g/g and 0.6 g/g for potato and anchote, respectively. WSI measures the number of free molecules leached out from the starch granules in addition to excess water and thus reflects the extent of starch degradation [41].

The mean viscosity values were observed as 235.3 mPa s and 241.7 mPa s for potato and anchote starches, respectively. The higher viscosity was observed for the anchote starch. The results of pH for starch samples were reported as 6.0 and 6.1 for potato and anchote starch, respectively. The pH values of the starch in water suspension are important because some functional properties like solubility and emulsion properties are highly affected by change in pH [37]. The minimum solubility for most of the starches was observed at pH 4.0 and 6.0. The high solubility of starches under highly acidic conditions (pH 2.0) may be due to their enhanced hydrophilic character and partial hydrolysis. Under alkaline conditions, the starch may undergo to partial gelatinization, thus resulting starch results in higher solubility at pH 10.0 [23]. In general, both potato and anchote starches can be used to enhance viscosity and thicken, stabilize, and enhance the mouth feel and smoothness of foods.

### 3.2. Proximate Composition of Starch

Analysis of variance showed significant difference (*P* ≤ 0.05) among potato and anchote starches in the proximate composition (Table 2) with the exception of crude protein and fat content. The moisture contents of the potato and anchote starches were observed as 5.3% and 10.3%, respectively, which were within the safe limit for storage requirements for starches [42]. The anchote starch had the higher moisture content than the potato starch and this may be attributed to the variation in methods used for the starch extraction. These results are higher than previously reported by Abera et al. [22]. The recommended moisture content for storing commercial starch is 10–12%. The moisture content > 12% encourages the microbial growth and induces degradative biochemical reactions leading to spoilage of starches in storage [40]. Starches with lower moisture content are less prone to microorganism degradation, making amenable for utilization in industries like the pharmaceutical industry. Moisture contents lower than 10% are required for starch incorporation into low-density polyethylene matrix in the production of biodegradable products [28, 36].

The crude protein contents of potato and anchote starches were 0.30% and 0.4%, respectively. The protein contents of starches were similar as previously reported by Abera et al. [22]. Low protein content is may be due to the sources, usually, roots and tubers that do not contain endosperm protein which could affect the purity and crystal structure of the starches. Low protein content of the starches adversely affects the physicochemical properties of the starches [28]. The fat contents in starches were observed as 0.2% and 0.30% for potato and anchote, respectively. These values were in agreement with the report of Parmar et al. [43] and Abera et al. [22]. The ash contents were observed as 0.3% and 1.1% for potato and anchote starch, respectively. Earlier studies reported ash contents similar with the present study results of potato and anchote starches [22, 43, 44]. The low ash content is a quality indicator for a good quality starch [28]. The ash content below 0.5% is recommended for higher-grade industrial starches [45].

### 3.3. Amylose and Amylopectin Contents of Potato and Anchote Starch

The total starch contents in potato and anchote starches were observed as 89.7% and 76.3%, respectively. The value of potato starch content was similar with the value reported by Abera et al. [22]; in contrast, a lower value was recorded for anchote starch contents.

The amylose contents in potato and anchote starches were 25.7% and 15.8%, respectively. Similar amylose content in potato starch has been reported by Sanchez-González et al. [31] and Vafina et al. [46]. The amylose content of starch determines its properties (such as water binding capacity, thickening, and gelling) and dictates most of its end uses. With the determined amylase contents, both the starches can successfully applied in industries as thickeners and binders [28]. High amylose content starch granules had low lipid and ash contents. Lipids bind to amylose molecules lead to the formation of an amylose-lipid complex that competes with iodine to form a complex [40, 47].

The amylopectin contents in potato and anchote starches were 75.8% and 84.5%, respectively. Similar amylose content in potato starch has been reported by Sanchez-González et al. [31]. The relative amounts of amylose and amylopectin are known to influence both nutritional and technological properties of starch such as susceptibility to enzymatic hydrolysis, gelling, and pasting behavior, which could be of biotechnological importance [48].

### 3.4. Effect of Starch Addition on Physicochemical Properties of Pineapple Juice

The results on the physicochemical properties of pineapple juice as a function of storage time are given in Table 3. It can be observed that the effect of all main factors and their interactions was significantly affected on sedimentation of juice at *P* ≤ 0.05. There was a significant difference observed due the effect of starch type and concentration on sedimentation of pineapple juice after 7 days of storage. The results showed that the highest sedimentation was observed in control samples (51.33%) stored for 15 days, whereas no (0%) sedimentation was observed on the initial day of storage. In terms of starch type, the highest sedimentation (31.17%) was observed in 5% potato starch-added samples while the lowest sedimentation (6.50%) was observed in 5% anchote starch-added pineapple juice samples stored for 15 and 7 days, respectively.

Pineapple juices with potato starch showed higher sedimentation than juice with anchote starch. The control samples showed highest degree of sedimentation as compared to starch-added samples during the storage time. The sedimentation and phase separation were observed in the first 24 h of stored control samples; it is a common behavior of some fruit juices [11]. After the first day of storage, both starch-added and control samples showed particle sedimentation as an increase in storage durations. The major sedimentation changes were happened in the first 7 days of the storage. Moreover, it is interesting to note that the control samples showed quicker sedimentation than the starch-added samples. This may be due to the aggregation of particles, which causes quick sedimentation.

As the storage time increased, the sedimentation also increased; in contrast, as the starch concentration increased, the sedimentation decreased in the first 7 days of storage while increased after 7 days of storage. This may be due to the increase in concentration of starch that leads to the settlement of cloud. It has been reported that incorporation of starch with higher concentrations leads to a higher electrolyte concentration, resulting in salting out of the starch [14]. Pineapple juice with anchote starch was showing less sedimentation than pineapple juice with potato starch.

The sedimentation decreased significantly (*P* ≤ 0.05) with increasing levels of starch incorporation into the pineapple juice as compared with the control samples. This may be attributed to the rise in the juice viscosity due to the addition of the starch. In general, the sedimentation of juices varied significantly (*P* ≤ 0.05) with increasing levels of starch addition and storage durations. As given by the Stokes law, particle sedimentation velocity is inversely proportional to the dispersed medium viscosity [11, 49]. Therefore, the increase in viscosity prevents aggregation of particles that causes a reduction in particle size in the suspension. This can be attributed to the greater stability of the starch-added samples, and starch can be seen as an important tool in preventing the juice sedimentation.

Analysis of variance (Table 3) showed that the effect of interactions and main effects was not significant on pH of pineapple juice at *P* ≤ 0.05. There was no significant difference (*P* ≤ 0.05) that was observed due to the effect of starch type and concentration on pH of pineapple juice as a function of storage durations. As the results have showed, the highest pH (3.49) value was observed in 1% anchote starch-added juice at the initial day, while the lowest (2.0) was determined in 5% potato starch-added pineapple juice after 15 days of storage. Besides, depending on the starch type, the highest pH was shown in 1% anchote starch-added samples, while the lowest was observed in 5% potato starch-incorporated juice (Table 3). Pineapple juices added with potato starch have shown higher pH than juice with anchote starch. The statistical analysis implied that as the storage time increases, the pH level in pineapple juice was considerably decreased due to the production of organic acids during storage because of fermentation. However, pH remained almost unchanged with the increasing level of starch and type of starch. Mahomud et al. [50] reported an increase in pH of tomato juice with the addition of starch which was deviated from the findings of this work. The pH results observed in this study were similar to the value reported by Nadzirah et al. [10]. In general, incorporating the starch had no significant effect on the mean value of the pH of pineapple juice.

The effect of interactions and main effects was significant (*P* ≤ 0.05) in TA of pineapple juice as illustrated in Table 3. A significant difference in TA was observed by the effect of starch type. However, there was no significant effect observed (*P* ≤ 0.05) due to starch concentration on TA of pineapple juice except on the 7^th^ day of storage in both the starch type additions. The highest TA (0.52%) was observed in 3% potato starch-added pineapple juice samples after 15^th^ days of storage. In contrast, the lowest TA (0.25%) is in 5% anchote starch-added pineapple juice samples at the initial day. In terms of starch type, the highest TA was observed in 3% potato starch, whereas the lowest is in 5% anchote starch-added pineapple juice. The juices with potato starch showed slightly higher TA than anchote starch-added juices. However, it remained almost unchanged with the increasing concentration of starch.

The ANOVA showed that pineapple juices exhibited a slight increase in the TA over storage time and were significantly varied (*P* ≤ 0.05). The TA results of this study were in agreement with reports of Ghafari and Ansari [33] and Shamsudin et al. [3]; they reported a significant relationship between the gradual increase in acidity of pineapple juice during the storage and the amount of organic acid produced. In this study, generally, the results of TA increased as the storage duration increased, but it remained almost unchanged with starch type and increasing levels of starch concentration.

The ANOVA showed that the effect of interactions was not significant on TSS of pineapple juice at *P* ≤ 0.05. Besides, there was no significant effect (*P* ≤ 0.05) on TSS due to starch type except on the 15^th^ day of pineapple juice has stored. There was a significant effect (*P* ≤ 0.05) on TSS of pineapple juice during storage by starch concentration except the 15^th^ day of storage. The results showed that the highest TSS was observed in 5% anchote starch (15.13%) at the initial day, while the lowest TSS (13.03%) was observed in 1% potato starch-added pineapple juice after the 15^th^ day of storage. In addition, in terms of starch type, the highest TSS was observed in the samples with 5% anchote starch addition, while the lowest was observed in 1% potato starch-incorporated juice. Pineapple juices with anchote starch showed a slightly higher TSS than juice with potato starch addition. However, there was no statistically significant difference; a slight increase in TSS was observed as starch concentration increased.

The analysis of variance revealed that TSS of pineapple juices slightly decreased over storage durations and were not significantly varied (*P* ≤ 0.05) between the treatments. At lower pH, juices are comparatively effluent in organic acids and result in low TSS content [51]. Besides, significant differences (*P* ≤ 0.05) were not observed on TSS by starch type and starch concentration. The results of TSS are similar to the values reported by Lu et al. [5], Ghafari and Ansari [33], and Shamsudin et al. [3].

There was no significant (*P* ≤ 0.05) difference observed due to the interactions effects on vitamin C content of pineapple juice, except at the initial day of storage. A significant difference (*P* ≤ 0.05) was not observed in vitamin C content of pineapple juice by the effect of starch type and starch concentration, except a significant effect by starch concentration was observed at the 7^th^ day of storage. The highest vitamin C concentration was observed in 5% potato starch-added juice sample (21.64 mg/100g) at the initial day, whereas the lowest vitamin C content (17.30 mg/100g) was determined in 1% potato starch-added pineapple juice after 15 days of storage. Similar results were observed due to the starch type with respect to the highest and lowest vitamin C content of pineapple juice. The vitamin C content of pineapple juice with potato and anchote starches was almost unchanged. The vitamin C content in starch-added and control pineapple juice samples was observed higher on initial day. Similarly, there was no significant statistical difference among the vitamin C composition of different samples on initial day. However, as the storage duration increases, the vitamin C content decreased in all the samples. This result was in agreement with reports of Rashima et al. [1], Hounhouigan et al. [52], and Wardy et al. [53]. The vitamin C content of pineapple juice decreases gradually with increase in storage time especially after 7 days of storage due to the oxidation reactions [30]. However, there was no significant difference (*P* ≤ 0.05) on vitamin C content. Besides, there was no difference (*P* ≤ 0.05) observed in vitamin C content of pineapple juice due to the starch concentration and type of starch.

The ANOVA showed that the viscosity of pineapple juice significantly (*P* ≤ 0.05) depends on interactions and main effects. The results showed that the highest viscosity (70 mPa s) was observed in 5% anchote starch-added pineapple juice at the initial day, while the lowest viscosity (10 mPa s) was observed in control juice after 15 days of storage. Moreover, in terms of starch type, the highest viscosity (70 mPa s) was shown in 5% anchote starch, whereas the lowest viscosity (19 mPa s) was observed in 1% potato starch-incorporated juice. Juices with anchote starch were observed for higher viscosity than juice with potato starch addition. As the concentration of the starch increased in the juice, the viscosity also increased. Besides, as the storage time increased, the viscosity of pineapple juice decreased significantly (*P* ≤ 0.05).

This tread may be attributed to the degree of starch polymerization. With a high degree of polymerization, the solution viscosity will be high. The results of the present study are in agreement with the findings of Shamsudin et al. [25]. Compared with starch type, anchote starch facilitated more viscous juice than potato starch. Lower concentration of starch addition facilitated pineapple juice with low viscosity, and as the storage duration increased, phase separation occurred in the product. This trend in this result corresponds to the theory of Tan [54]; increasing viscosity corresponds to the increase in the stability in turbidity [20]. Generally, in this study, it is observed that storage time, starch concentration, and type of starch affected the viscosity of pineapple juice significantly (*P* ≤ 0.05).

The statistical analysis showed that the effect of interactions and main effects was significant on the turbidity of pineapple juice at *P* ≤ 0.05. The results revealed that the highest turbidity (696.33 FTU) was observed in 5% anchote starch-added pineapple juice at the initial day, while the lowest turbidity (102 FTU) was observed in control juice after 15 days of storage. Besides, in terms of starch type, the highest turbidity (696.33 FTU) was observed in 5% anchote starch, whereas the lowest turbidity (152.33 FTU) was in 1% potato starch-incorporated juice. Pineapple juice with anchote starch showed higher turbidity than juice with potato starch-added samples. As the concentration of the starch increased in the juice, the turbidity increased significantly (*P* ≤ 0.05). Moreover, as the storage duration increased, the turbidity of pineapple juice decreased significantly (*P* ≤ 0.05). The major changes in the turbidity of the pineapple juice were taken place in the first 7 days of the storage. As storage time increased, cloudiness decreased with rise in the starch concentration. This can be attributed to the decrease in viscosity and increase in particle sedimentation which allows more light to transfer through the juice. The results of this study revealed that as the increase in starch concentration is from 1% to 5%, the pineapple juice cloudiness also increased due to the loss in viscosity and movement of particles in the system. The control juice showed a lesser degree of cloudiness as compared to the starch-added juice samples during the storage period.

The turbidity of the pineapple juice decreased significantly (*P* ≤ 0.05) with increase of storage duration as compared to the control samples. In addition, the turbidity of juices varied significantly (*P* ≤ 0.05) with increase of starch concentration and starch type. Moreover, all the juice samples showed cloudiness variation along the storage durations, reflecting the particle aggregation due to attractive forces. The juices with higher concentration of starch showed lower variation in turbidity than the other samples.

The cloud retention in pineapple juice was significantly improved by the addition of starch. As studies reported, juice cloudiness occurs mainly due to the suspension of solid particles [55]. There is a reverse relation between the turbidity and amount of sediment in the juice. Consequently, the turbidity decreased as separation of the product increased. In this study, the result showed the starch-added pineapple juice samples followed this reversible relation. Moreover, the cloud stabilizing effect of starch could be attributed to the strong water binding characteristics of the starch, which forms a hydrate shell around the cloud particles that adjusts the density of the cloud particles with the serum [14].

According to the Stokes law, the larger-sized particles are easier to precipitate. A good thickener can prevent the formation of large polymers. The difference in absorbance values was mainly influenced by the particles that remained in suspension [15, 49].

### 3.5. Sensory Acceptability of Pineapple Juice

**Figure 1:**
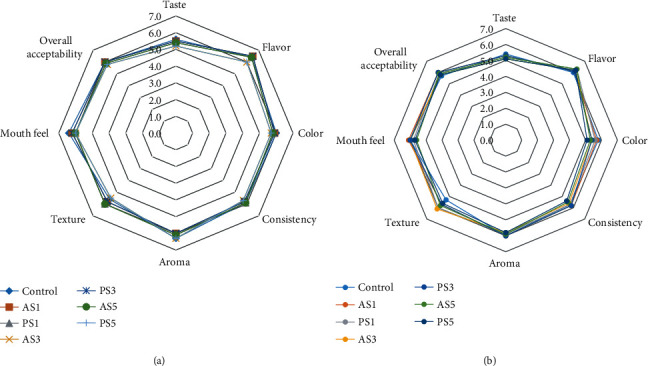
Sensory acceptability of pineapple juice. (a) Initial day. (b) Stored for 7 days. Control: pineapple juice (without starch); AS1: 1% anchote starch; AS3: 3% anchote starch; AS5: 5% anchote starch; PS1: 1% potato starch; PS3: 3% potato starch; PS5: 5% potato starch.

The results of sensory acceptability of pineapple juices as a function of storage time are presented in Figure 1. In this work, the results for sensory acceptability were presented for the first day and 7^th^ day of the storage. It can be observed that the effect of interactions and all main effects was not significant on sensory characteristics of pineapple juice at *P* ≤ 0.05. The results of sensory testing for taste, flavor, color, consistency, aroma, texture, mouth feel, and overall acceptability showed no significant difference (*P* ≤ 0.05) between the juices containing potato and anchote starch. Therefore, the addition of starch did not affect these sensory parameters of the pineapple juice. However, pineapple juice with starch addition showed better acceptability than pineapple juice with no starch, in terms of appearance, texture, and mouth feel. As the starch concentration increased in the juice, the panelists are given sensory score from like very much to like moderately for all evaluated sensory attributes. The type and amount of starch had no significant effect on sensory acceptability of pineapple juice. Similar studies have been done by Akkarachaneeyakorn and Tinrat [20] and Lv et al. [15] on the effect of hydrocolloids on sensory evaluation of mulberry fruit juice and orange juice, respectively. Besides, storage time also had no significant effect on sensory evaluation of pineapple juice even though there was a slight reduction in the sensory acceptability scores. The acceptability trend was steady to some extent with small changes for few sensory attributes. At high concentration of starch addition, the pineapple juice becomes sticky; therefore, the starch that added to pineapple juice should keep the original taste of juice.

Subsequently, aroma, flavor, and mouth feel of juice are the most important factors that affect the pineapple juice acceptability; all consumers like pineapple juice with good flavor, mouth feel, and aroma. In terms of the texture, it is also an important factor; generally, soft texture is easy to accept by humans. Overall, acceptance of the juices with starch is important because consumers are not interested in consuming pineapple juice which is not in good appearance.

### 3.6. Shelf Life Determination of the Pineapple Juice

**Figure 2:**
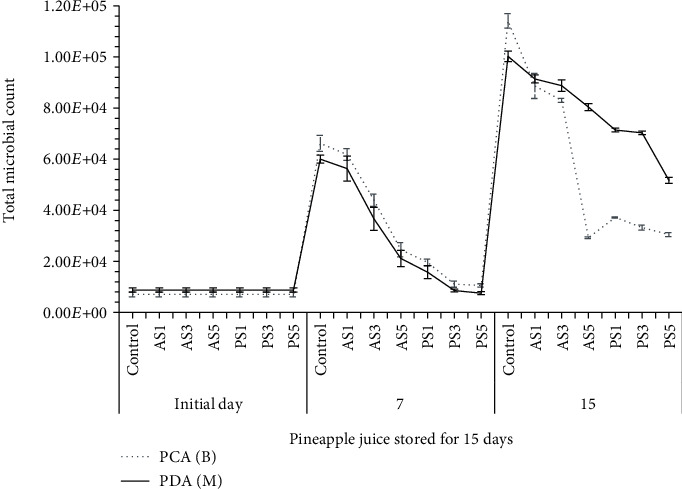
Microbial load of pineapple juice stored for 15 days. Control: pineapple juice (without starch); AS1: 1% anchote starch; AS3: 3% anchote starch; AS5: 5% anchote starch; PS1: 1% potato starch; PS3: 3% potato starch; PS5: 5% potato starch; PCA (B): total bacterial count; PDA (M): total fungi count.

The effect of storage time, type, and amount of starch on the total viable bacterial and fungal counts of pineapple juice during storage is illustrated in Figure 2. It can be observed that the effect of interactions and main effects was not significant on microbial counts of pineapple juice at *P* ≤ 0.05 at the initial day of storage. However, a significant effect (*P* ≤ 0.05) was observed due to the interactions and main effects after the initial storage day on microbial counts of pineapple juice. The results revealed that the highest bacterial count was observed in control juice (1.14 × 10^5^ cfu/ml) after 15 days of storage, whereas the lowest bacterial count (7.04 × 10^3^ cfu/ml) was observed in 5% anchote starch-added pineapple juice at the initial day. Besides, the highest fungal count was observed in control juice (1.00 × 10^5^ cfu/ml) samples at the initial day, while the lowest fungal count (8.77 × 10^3^ cfu/ml) was observed in 3% and 5% anchote starch-added pineapple juice after 15 days of storage. In terms of starch type, the highest bacterial count was shown in 1% anchote starch, whereas the lowest is in 5% anchote starch-incorporated juice. Similarly, the highest and lowest fungal counts were observed in 1% and 5% anchote starch-added samples, respectively. Juices with anchote starch were observed for higher bacterial and fungal counts than juice with potato starch added. As the starch concentration increased in the juice, the bacterial and fungal counts decreased significantly (*P* ≤ 0.05) except between 1% and 3% starch concentrations. Higher bacterial and fungal counts were observed in control samples than the starch-added pineapple juice during the storage. This can be ascribed to strong water binding characteristics of the starch, which combine with water to form viscous solutions which restrict access of water for microbial growth [46]. The pineapple juice with 5% of potato and anchote starch generally showed decreased microbial count than other treatments.

The results revealed that the total bacterial and fungal count in all pineapple juices increased throughout the storage time. However, increased starch concentration had a significant decreasing effect on the microbial content compared with the control juice during storage. In general, control sample showed remarkably high microbial loads during the period of storage and this may be possibly a major cause of spoilage commonly experienced by the producers of this product [9]. It is evident from this study that the starch-added juice samples had lower microbial loads when compared with the control juice.

## 4. Conclusions

The results of this study revealed that addition of potato and anchote starch significantly improved the cloud stability of the pineapple juice as compared to the control. The findings showed that starch type and starch concentration levels had significantly influenced some of the physicochemical parameters (turbidity, viscosity, and sedimentation) and microbial content of the pineapple juice in comparison to the control. Moreover, storage time had significant influenced on turbidity, viscosity, sedimentation, and microbial counts of the stored pineapple juice. As starch concentration in the juice increased, minimum influence on vitamin C content, TSS, TA, and pH of juice was observed. With increasing in storage time, turbidity, viscosity, TSS, pH, and vitamin C content of juice decreased, whereas sedimentation, TA, and microbial counts were increased. Adding starch with different concentrations had not significantly influenced the sensory acceptability of pineapple juice, even though there were some changes observed for some sensory attributes. In general, the findings showed that addition of starch with different concentrations had significantly influenced some physicochemical and shelf life of pineapple juice as a function of time.

The result showed that pineapple juice with 5% anchote starch had better cloud stability during storage period. This is because of product exhibited less pulp sedimentation and high viscosity and turbidity. Generally, addition of 5% starch helped in maintaining the cloud stability, reduction in microbial load, and no influence on the sensory acceptability of the pineapple juice over a period of 15 days.

## Figures and Tables

**Table 1:** Functional and physicochemical properties of potato and anchote starch.

Starch sample	Bulk density (g/cm^3^)	WAC (g/g)	WSI (g/g)	Swelling power (%)	Viscosity (mPa s)	pH
Anchote	1.4 ± 0.1^A^	1.1 ± 0.2^A^	0.6 ± 0.1^A^	5.5 ± 0.2^A^	241.7 ± 1.5^A^	3.3 ± 0.2^A^
Potato	1.5 ± 0.1^A^	1.4 ± 0.2^B^	1.2 ± 0.2^B^	4.9 ± 0.1^B^	235.3 ± 1.5^B^	3.4 ± 0.1^A^
*P* value	≤0.124	≤0.026	≤0.005	≤0.001	≤0.007	≤0.152

Values are presented as the means ± standard deviations (*n* = 3). Means with different superscript letters within a column differ significantly, *P* ≤ 0.05. WAC: water absorption capacity; WSI: water solubility index.

**Table 2:** Proximate composition and amylase and amylopectin contents of starch from anchote and potato tubers.

Starch sample	Moisture (%)	Ash (%)	Fat (%)	Starch (%)	Protein (%)	Amylose (%)	Amylopectin (%)
Anchote	10.3 ± 0.6^A^	1.1 ± 0.1^A^	0.3 ± 0.1^A^	76.3 ± 0.6^A^	0.4 ± 0.1^A^	15.8 ± 0.8^A^	84.5 ± 0.5^A^
Potato	5.3 ± 0.6^B^	0.3 ± 0.1^B^	0.2 ± 0.1^A^	89.7 ± 0.6^B^	0.3 ± 0.1^A^	25.7 ± 0.6^B^	75.8 ± 0.8^B^
*P* value	≤0.001	≤0.001	≤0.206	≤0.001	≤0.116	≤0.001	≤0.001

Values are presented as the means ± standard deviations (*n* = 3). Means with different superscript letters within a column differ significantly at *P* ≤ 0.05.

**Table 3:** Physicochemical properties of pineapple juice samples added with different types of starch with different concentrations stored for 15 days.

Storage time (days)	Juice sample	Sedimentation (%)	TSS (%)	pH	Viscosity (mPa s)	Turbidity (FTU)	TA (%)	Vitamin C (mg/100g)
Initial day	Control	—	14.30 ± 0.78^AB^	3.47 ± 0.11^A^	24.0 ± 1.0^F^	432.67 ± 1.15^G^	0.28 ± 0.02^AB^	21.57 ± 0.50^A^
	AS1	—	14.39 ± 0.43^AB^	3.49 ± 0.28^A^	39.33 ± 1.53^D^	481.33 ± 0.58^E^	0.27 ± 0.02^AB^	21.25 ± 0.45^A^
	AS3	—	14.52 ± 0.66^AB^	3.46 ± 0.12^A^	60.33 ± 1.53^B^	555.33 ± 1.15^C^	0.26 ± 0.01^B^	21.23 ± 0.08^A^
	AS5	—	15.13 ± 0.57^A^	3.44 ± 0.06^A^	70.0 ± 1.0^A^	696.33 ± 2.08^A^	0.25 ± 0.01^B^	21.50 ± 0.36^A^
	PS1	—	14.34 ± 0.62^AB^	3.42 ± 0.06^A^	33.33 ± 1.15^E^	462.67 ± 1.15^F^	0.27 ± 0.01^AB^	21.48 ± 0.18^A^
	PS3	—	14.36 ± 0.44^AB^	3.43 ± 0.05^A^	49.33 ± 0.58^C^	496.67 ± 0.58^D^	0.27 ± 0.01^AB^	21.53 ± 0.45^A^
	PS5	—	14.87 ± 0.70^A^	3.40 ± 0.07^A^	59.67 ± 1.53^B^	569.67 ± 1.53^B^	0.26 ± 0.02^B^	21.64 ± 0.67^A^

Day 7	Control	31.0 ± 1.0^A^	14.22 ± 0.29^A^	3.22 ± 0.13^A^	19.33 ± 1.15^F^	225.33 ± 1.53^FG^	0.32 ± 0.01^C^	20.47 ± 0.37^A^
	AS1	16.83 ± 0.76^C^	14.32 ± 0.24^A^	3.20 ± 0.10^A^	30.67 ± 1.15^E^	271.67 ± 1.53^D^	0.33 ± 0.02^BC^	20.23 ± 0.24^A^
	AS3	12.33 ± 0.58^D^	14.45 ± 0.19^A^	3.21 ± 0.14^A^	43.0 ± 1.0^C^	406.33 ± 1.53^C^	0.34 ± 0.01^ABC^	20.78 ± 0.48^A^
	AS5	6.50 ± 0.50^F^	15.01 ± 0.55^A^	3.20 ± 0.20^A^	67.67.0 ± 58^A^	595.33 ± 1.53^A^	0.36 ± 0.02^AB^	20.87 ± 0.76^A^
	PS1	20.83 ± 1.26^B^	14.30 ± 0.19^A^	3.24 ± 0.17^A^	29.0 ± 1.73^E^	222.67 ± 1.53^G^	0.35 ± 0.02^ABC^	20.11 ± 0.12^A^
	PS3	14.0 ± 1.0^D^	14.34 ± 0.31^A^	3.16 ± 0.07^A^	40.0 ± 1.0^D^	238.0 ± 1.0^E^	0.36 ± 0.02^ABC^	20.17 ± 0.19^A^
	PS5	9.50 ± 0.87^E^	14.68 ± 0.81^A^	3.19 ± 0.12^A^	64.67 ± 1.53^B^	424.67 ± 1.15^B^	0.38 ± 0.01^A^	20.47 ± 0.20^A^

Day 15	Control	51.33 ± 0.58^A^	13.15 ± 0.06^A^	2.02 ± 0.04^A^	10.0 ± 1.0^G^	102.0 ± 1.0^G^	0.49 ± 0.01^BC^	17.77 ± 0.08^A^
	AS1	18.50 ± 0.50^E^	13.43 ± 0.34^A^	2.03 ± 0.06^A^	25.0 ± 1.0^E^	172.33 ± 2.08^E^	0.47 ± 0.02^C^	17.35 ± 0.35^A^
	AS3	22.0 ± 1.0^CD^	13.75 ± 0.66^A^	2.08 ± 0.10^A^	35.33 ± 0.58^C^	226.33 ± 2.31^C^	0.51 ± 0.01^ABC^	17.40 ± 0.26^A^
	AS5	29.83 ± 0.76^B^	13.97 ± 0.55^A^	2.04 ± 0.05^A^	64.33 ± 1.53^A^	377.33 ± 1.53^A^	0.50 ± 0.01^ABC^	17.63 ± 0.28^A^
	PS1	20.17 ± 1.04^DE^	13.03 ± 0.57^A^	2.03 ± 0.04^A^	19.0 ± 1.0^F^	152.33 ± 2.08^F^	0.48 ± 0.01^ABC^	17.30 ± 0.17^A^
	PS3	24.0 ± 1.0^C^	13.23 ± 0.40^A^	2.02 ± 0.02^A^	32.0 ± 1.0^D^	206.0 ± 2.65^D^	0.52 ± 0.03^A^	17.80 ± 0.56^A^
	PS5	31.17 ± 0.76^B^	13.69 ± 0.88^A^	2.00 ± 0.01^A^	55.0 ± 1.0^B^	349.03 ± 0.15^B^	0.51 ± 0.02^AB^	17.85 ± 0.18^A^

*P* value								

Starch type∗concentration	Initial day	—	≤0.185	≤0.54	≤0.001	≤0.001	≤0.098	≤0.032
Starch type		—	≤0.262	≤0.229	≤0.001	≤0.001	≤0.065	≤0.26
Starch concentration			≤0.003	≤0.148	≤0.001	≤0.001	≤0.009	≤0.457

Starch type∗concentration	Day 7	≤0.001	≤0.962	≤0.314	≤0.001	≤0.001	≤0.052	≤0.686
Starch type		≤0.001	≤0.125	≤0.885	≤0.001	≤0.001	≤0.001	≤0.074
Starch concentration		≤0.001	≤0.012	≤0.177	≤0.001	≤0.001	≤0.21	≤0.005

Starch type∗ concentration	Day 15	≤0.001	≤0.839	≤0.54	≤0.001	≤0.001	≤0.034	≤0.216
Starch type		≤0.001	≤0.06	≤0.229	≤0.001	≤0.001	≤0.053	≤0.072
Starch concentration		≤0.001	≤0.198	≤0.148	≤0.001	≤0.001	≤0.006	≤0.18

Values are presented as the means ± standard deviations (*n* = 3). Means with different superscript letters within a column differ significantly at *P* < 0.05. Control: pineapple juice without starch; AS1: 1% anchote starch; AS3: 3% anchote starch; AS5: 5% anchote starch; PS1: 1% potato starch; PS3: 3% potato starch; PS5: 5% potato starch.

## Data Availability

The data used to support the findings of this study are included within the article.
